# Recurrence and Prognostic Value of Asymptomatic Spinal Cord Lesions in Multiple Sclerosis

**DOI:** 10.3390/jcm10030463

**Published:** 2021-01-26

**Authors:** Camilla Ostini, Francesca Bovis, Giulio Disanto, Paolo Ripellino, Emanuele Pravatà, Rosaria Sacco, Giovanna Padlina, Maria Pia Sormani, Claudio Gobbi, Chiara Zecca

**Affiliations:** 1 Multiple Sclerosis Center, Department of Neurology, Neurocenter of Southern Switzerland, Ospedale Civico, Via Tesserete 46, 6903 Lugano, Switzerland; camilla.ostini@bluewin.ch (C.O.); giulio.disanto@eoc.ch (G.D.); paolo.ripellino@eoc.ch (P.R.); rosaria.sacco@eoc.ch (R.S.); giovanna.padlina@eoc.ch (G.P.); claudio.gobbi@eoc.ch (C.G.); 2 Department of Health Sciences, University of Genova, 16132 Genova, Italy; francesca.bovis@gmail.com (F.B.); mariapia.sormani@unige.it (M.P.S.); 3 Department of Neuroradiology, Neurocenter of Southern Switzerland, 6900 Lugano, Switzerland; emanuele.pravata@eoc.ch; 4 IRCCS, Ospedale Policlinico San Martino, 16132 Genova, Italy; 5 Faculty of Biomedical Sciences, Università della Svizzera Italiana, Via Buffi 13, 6900 Lugano, Switzerland

**Keywords:** multiple sclerosis, spinal lesions, asymptomatic, prediction, MRI

## Abstract

Spinal magnetic resonance imaging (MRI) is currently not recommended for the routine monitoring of clinically stable multiple sclerosis (MS) patients. We aimed to investigate the occurrence of asymptomatic spinal lesions (a-SL) in clinically stable MS patients, and their association with clinical and radiological outcomes, including the recurrence of spinal lesions. The hospital MS registry was searched for clinically stable MS patients (no relapses, no disability progression) with spinal MRIs performed at T1 (baseline) and T2 (9–36 months after T1). Information on relapses, disability and new brain/spinal MRI lesions at T3 (≥6 months after T2) was collected and analyzed. Out of 300 MS patients, 45 showed a-SL between T1 and T2. The presence of a-SL was not associated with the subsequent occurrence of relapses or disability progression at T3, but did correlate with the risk of new brain (rate ratio (RR) = 1.63, 95% CI = 1.16−2.25, *p* = 0.003) and recurrent spinal lesions (RR = 7.28, 95% CI = 4.02–13.22, *p* < 0.0001). Accounting for asymptomatic brain lesions (a-BL), the presence of either a-BL or a-SL was associated with subsequent risk for new brain (OR = 1.81, 95% CI = 1.25–2.60, *p* = 0.001) or spinal (RR = 2.63, 95% CI = 1.27–5.45, *p* = 0.009) lesions. Asymptomatic spinal demyelinating lesions occurred in 15% of clinically stable MS patients within a median period of 14 months and conferred an increased risk of future radiological activity at the brain and spinal level.

## 1. Introduction

Multiple sclerosis (MS) is the most common inflammatory disease of the central nervous system in young adults leading to long-term disability, with spinal cord involvement being one of its most relevant determinants [1]. Lesions in the spinal cord are often located in clinically eloquent areas with tightly packed fibers, and consequently, fewer compensatory capacities, resulting in an increased risk of disability [2].

According to current MAGNIMS guidelines [3], spinal imaging is only indicated in case of spinal symptoms at clinical presentation in the differential diagnosis between MS and other neuroinflammatory conditions, or in case of radiologically isolated syndrome (RIS) for its prognostic value. Instead, spinal magnetic resonance imaging (MRI) is not recommended for the routine monitoring of clinically stable MS patients [4,5,6], mainly because of technical difficulties, limited MRI access, costs, and the belief that clinically silent lesions in the spinal cord are unlikely [7]. Nevertheless, an increasing body of evidence documents that spinal demyelinating lesions can also occur asymptomatically [8,9,10,11,12]. From our previous study of a group of 103 relapsing remitting MS patients, it emerged that a-SL occurred in approximately 25% of individuals over a median follow up of 17 months, even in the absence of new brain lesions, and both lesion types could be used to predict future clinical relapses [9]. Some studies also suggest that spinal involvement tends to recur in MS [13]. Despite this, it is currently debated whether detection of a-SL could be used as a surrogate marker of disease activity and treatment response, and therefore, whether spinal MRI should be included in the routine monitoring of MS patients.

This study aimed to investigate the frequency, recurrence, and the clinical and radiological prognostic value of a-SL in a large group of relapsing and progressive MS patients.

## 2. Materials and Methods

### 2.1. Study Population 

We queried the registry of our Multiple Sclerosis Center in May 2019 to identify MS patients who underwent a spinal 3.0 T MR imaging during their follow up. This registry was initiated in October 2007 and prospectively collects demographic, social, clinical, and radiological features of more than 90% of all the MS patients of southern Switzerland. All patients previously gave their informed consent for their data to be included in the MS registry and used for scientific purposes. The Ethics Committee of Canton Ticino approved this research project (Ref. CE 2911). 

### 2.2. Inclusion Criteria and Study Design

**Figure 1:**
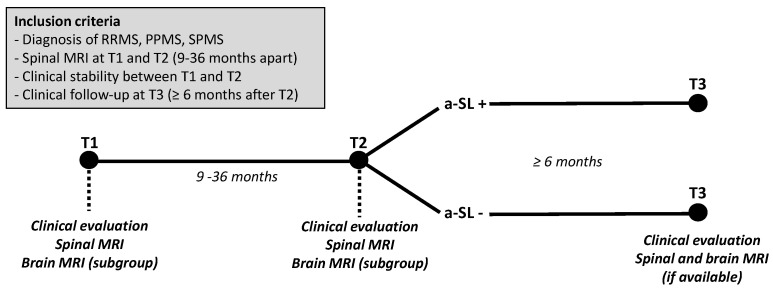
Inclusion criteria and study design. Spinal MRIs are performed at T1 (baseline) and T2 (9–36 months after T1) in clinically stable multiple sclerosis (MS) patients. When available, information on brain MRIs performed at T1 (±60 days) and at T2 (±60 days) were also collected and used for sensitivity analyses. Patients with vs. without asymptomatic spinal lesions (a-SL) at T2 are then compared in terms of clinical and radiological outcomes at T3 (>6 months after T2). RRMS: relapsing-remitting multiple sclerosis; PPMS: primary progressive multiple sclerosis; SPMS: secondary progressive multiple sclerosis.

Among patients diagnosed with relapsing-remitting multiple sclerosis (RRMS), primary progressive multiple sclerosis (PPMS) or secondary progressive multiple sclerosis (SPMS) according to either 2010 or 2017 revised McDonald criteria [14,15], we selected those having received spinal MRI at baseline (T1), with repeated spinal MRI at T2 (i.e., 9–36 months after T1), with proven clinical stability between T1 and T2 (absence of relapses and absence of disability progression as measured by the Expanded Disability Status Scale (EDSS) score), and with a clinical follow-up (T3) visit of at least 6 months after T2. When available, information on brain MRIs performed at T1 (±60 days) and at T2 (±60 days) were collected and used for sensitivity analyses. Brain and spinal MRIs performed during follow-up at least 6 months after T2 were also considered for analyses. The study design is presented in Figure 1. Patients with incomplete clinical and/or radiological data, and those with a diagnosis of clinically isolated syndrome (CIS) or radiologically isolated syndrome (RIS) [16] were excluded.

### 2.3. Clinical Assessment

All patients in our MS registry undergo a standardized protocol of clinical follow-up, including a detailed neurological examination with EDSS assessment every 6 months and within two weeks in cases of suspected relapse. All neurologists treating patients included in the registry are certified at www.neurostatus.net. 

A “relapse” was defined—according to widely accepted guidelines [14]—as newly developing neurological symptoms or the worsening of pre-existing neurological dysfunctions lasting for a minimum of 24 h in the absence of fever or infections and occurring at least 30 days after the preceding episode. “Disability progression” was defined as an increase by ≥1 point in EDSS score in cases of a baseline EDSS of ≤ 5.5, or ≥ 0.5 points in cases of a baseline of EDSS ≥ 6.0, confirmed 6 months apart. 

For patients fulfilling the inclusion criteria, gender, age, and disease course at inclusion, disease duration, EDSS at T1, T2, and T3, number of relapses in the 2 years prior to T1, and between T2 and T3 (for RRMS) data were collected. 

### 2.4. Neuroimaging Assessment 

All spinal and brain MRIs were acquired using three identical Tesla scanners from Siemens (Skyra, Erlangen, Germany), applying the same routine protocol (Supplementary Material). The following data were collected: number of new cervical and thoracic lesions at T2 (compared to T1) and at T3 (compared to T2), and number of new brain demyelinating lesions at T2 (±60 days) vs. T1, and at T3 (±60 days) vs. T2. The new spinal and brain demyelinating lesions occurring during clinical stability (between T1 and T2) were reported as a-SL and asymptomatic brain lesions (a-BL), respectively.

### 2.5. Study Objectives

The study objectives were as follows: to investigate the occurrence of a-SL in clinically stable MS patients, and their individual role, as well as in combination with a-BL, in predicting subsequent clinical (relapses and disability progression) and radiological (new brain and spinal demyelinating lesions) outcomes.

### 2.6. Statistical Analysis

Continuous and ordinal variables were described by median and interquartile range (IQR). Categorical variables were described by counts and percentages. Differences in the distribution of these variables were tested for statistical significance using the chi-square tests or Fisher’s exact test for categorical variables and using the Mann–Whitney U-test for continuous variables, as appropriate. 

Cox regression analysis was used to test the association of a-SL and a-BL between T1 and T2 as well as demographic and clinical characteristics with time to first relapse (TTFR) and time to disability progression. Poisson regression analysis was used to test the association of the same factors with the annualized relapse rate (ARR) and the annualized new MRI lesion rate. Only factors significantly associated with the outcome in univariate analyses (with a *p* value < 0.05) were included in the multivariable model with a stepwise procedure (*p* for inclusion < 0.05). SAS 9.3 (Institute Inc., Cary, NC, USA) and R software (version 3.5.0) were used for the analysis.

## 3. Results

**Figure 2:**
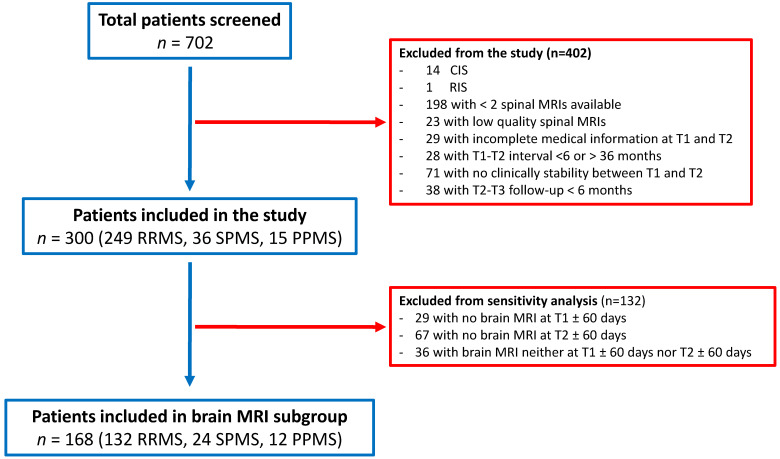
Flow chart of study population. A total of 702 MS patients were screened, 300 fulfilled the inclusion criteria and were therefore considered for analysis. A total of 168 patients also had a brain MRI available within ±60 days from T1 and T2 (brain MRI subgroup).

A total of 702 MS patients were screened from the registry, 300 patients fulfilled the inclusion criteria resulting in them being eligible for analysis (249 RRMS, 36 SPMS, 15 PPMS, Figure 2). Their baseline characteristics are reported in Table 1. Forty-five of the 300 (15.0%) patients had a-SL at T2 vs. T1 (RRMS = 38; SPMS = 3; PPMS = 4; cervical = 25; thoracic = 20) and all a-SL were negative for gadolinium enhancement. The median intervals between T1 and T2 and between T2 and T3 were 14.30 (12.00–20.83) and 31.07 (17.14–49.99) months, respectively. 

### 3.1. Disability Progression

At T3 the median EDSS was 2.5 (range 0–7.5) and 59 patients had disability progression as compared to T2. Out of the 45 patients with a-SL at T2, six (13.3%) had an increased EDSS at T3. Out of the 255 patients without a-SL at T2, 53 (20.8%) had an increased EDSS at T3. The univariate analysis showed that progressive disease course (compared to RRMS) was the only variable associated with an increased risk of EDSS progression (hazard ratio[HR] = 4.33 (95% CI = 2.08‒9.02) for PPMS and HR = 2.63 (95% CI = 1.41‒4.89) for SPMS; *p* < 0.0001 for both). Presence of a-SL between T1 and T2 and remaining variables (age at MS onset, disease duration, gender, baseline EDSS score, treatment history) were instead not associated with EDSS progression (Table 2).

### 3.2. Relapses

Among 249 RRMS patients, 26 (10.44%) experienced 40 relapses between T2 and T3. Out of the 38 RRMS patients with a-SL at T2, 5 (13.2%) had a relapse between T2 and T3, with an ARR of 0.08 (0.04–0.06). Out of the 211 RRMS patients without a-SL at T2, 21 (9.9%) had a relapse between T2 and T3, with an ARR of 0.05 (0.03–0.07). In the univariate analysis, the ARR between T2–T3 was inversely associated with the baseline EDSS score (rate ratio (RR) = 0.69 (95% CI = 0.52‒0.91), *p* = 0.010). Presence of a-SL between T1 and T2 was not associated with either ARR or TTFR (Table 2).

### 3.3. New Brain Demyelinating Lesions

A total of 266 patients had a brain MRI performed at T3. Among these, 69 (25.94%) patients had new brain lesions at T3 vs. T2. According to the univariate analysis, the risk of developing a new brain lesion at T3 was positively associated with the occurrence of a-SL between T1 and T2, and negatively associated with age, male gender, disease duration, EDSS and progressive MS course (Table 3). The associations between the risk of new brain lesions at T3 and presence of a-SL at T2, age, male gender and EDSS remained statistically significant in the multivariate analysis (Table 3). The occurrence of brain lesions at T3 was approximately 1.6 times higher in those patients with vs. without evidence of a-SL between T1 and T2 (RR = 1.63, 95% CI = 1.16‒2.25, *p* = 0.003). 

### 3.4. New Spinal Demyelinating Lesions

A total of 242 patients had a spinal MRI performed at T3. Among these, 27 (11.16%) patients had new spinal lesions at T3 vs. T2. According to the univariate analysis, the risk of developing a new spinal lesion at T3 was positively associated with the occurrence of a-SL between T1 and T2, and negatively associated with disease duration and EDSS score (Table 3). The associations between risk of new spinal lesions at T3 and both presence of a-SL at T2 and EDSS remained statistically significant in the multivariate analysis (Table 3). Notably, the occurrence of spinal lesions at T3 was approximately seven times higher in those patients with vs. those without evidence of a-SL between T1 and T2 (RR = 7.28, 95% CI = 4.02‒13.22, *p* < 0.0001). 

### 3.5. Subgroup of Patients with Available Brain MRI at T1 and T2 

To further validate our findings, all previous analyses were repeated for the subgroup of 168 (132 RRMS, 24 SPMS, 12 PPMS) patients with not only spinal, but also available brain MRIs within ±60 days from T1 and T2 (brain MRI-subgroup). Their baseline characteristics are reported in Table 1. Out of these 168 patients, 26 (15.48%) had a-SL, 31 (18.45%) had a-BL, 48 (28.57%) had either a-SL or a-BL, and 17 (10.12%) had a-SL but not a-BL. The median interval between T2 and T3 was 37.28 (19.96–65.57) months. The occurrence of either a-SL or a-BL was not associated with risk of disability progression or relapses between T2 and T3 (Supplementary Tables S1 and S2).

**Figure 3:**
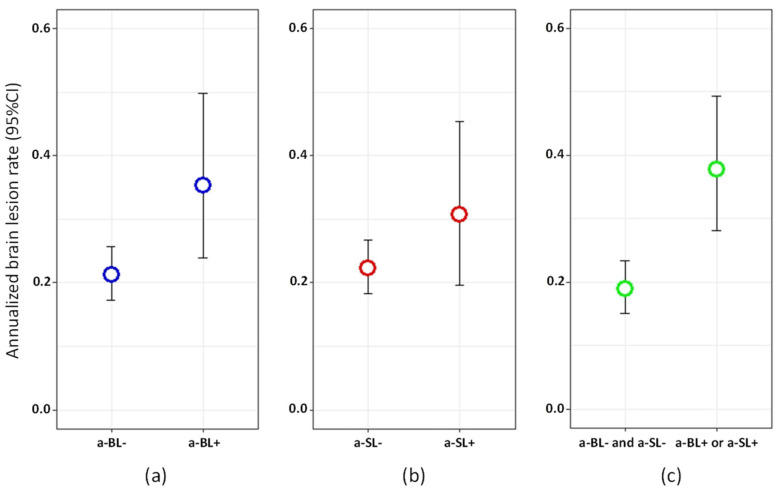
Annualized brain lesion rate between T2 and T3 in: (**a**) patients with vs. without asymptomatic brain lesions (a-BL) at T2; (**b**) patients with vs. without asymptomatic spinal lesions (a-SL) at T2; (**c**) patients with either a-BL or a-SL vs. neither a-BL nor a-SL at T2.

Among the patients with available follow-up MRIs, 42/154 (27.27%) had new brain lesions at T3 and 20/146 (13.70%) had new spinal lesions (12 at the cervical and nine at the thoracic level) at T3. According to the univariate analyses, the risk of developing a new brain lesion at T3 was higher in patients with a-BL at T2 (RR = 1.66, 95% CI = 1.08‒2.48, *p* = 0.016). The association appeared stronger in the presence of either a-BL or a-SL at T2 (RR = 2.00, 95% CI = 1.39‒2.84, *p* = 0.0001, Figure 3). Age at MS onset, as well as EDSS scores, were also inversely associated with risk of new brain lesions at T3 (Supplementary Table S3). When included in a multivariate model, only the presence of either a-BL or a-SL (OR = 1.81, 95% CI = 1.25‒2.60, *p* = 0.001) at T2, age and EDSS remained significantly associated with the risk of new brain lesions at T3.

**Figure 4:**
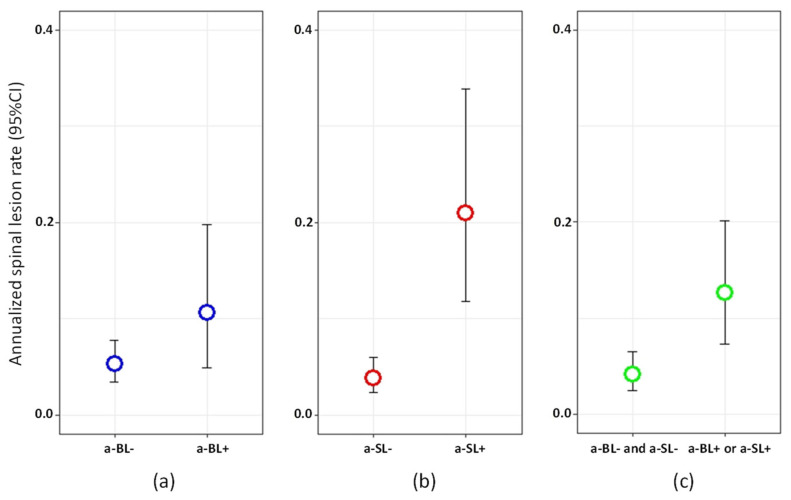
Annualized spinal lesion rate between T2 and T3 in: (**a**) patients with vs. without asymptomatic brain lesions (a-BL) at T2; (**b**) patients with vs. without asymptomatic spinal lesions (a-SL) at T2; (**c**) patients with either a-BL or a-SL vs. neither a-BL nor a-SL at T2.

According to the univariate analysis, the risk of developing a new spinal lesion at T3 was positively associated with the presence of a-SL at T2 (RR = 5.45, 95% CI = 2.64‒11.04, *p* < 0.0001), as well with the presence of either a-SL or a-BL at T2 (RR = 3.08, 95% CI = 1.51‒6.26, *p* = 0.002, Figure 4), and negatively associated with disease duration and EDSS (Supplementary Table S4). In the multivariate analysis, the presence of either a-SL or a-BL at T2 was retained in the model and was associated with a 2.6 increased occurrence of spinal lesions at T3 (RR = 2.63, 95% CI = 1.27‒5.45, *p* = 0.009).

## 4. Discussion

In our study, a-SL occurred in 15% of the clinically stable MS cohort—including relapsing and progressive patients—over a median period of 14 months. This figure was generally higher, ranging from 31.6 to 60%, in small studies published before the 2000s [17,18]. This heterogeneity likely reflects different study populations and designs, as well as sample sizes and MRI protocols. Additionally, in our previous study including 103 RRMS patients, we found that 25.2% had a-SL over a median period of 17 months [9]. In another cohort study, 33% of 135 new onset MS patients with spinal MRIs available at baseline had new spinal lesions after 2 years of follow up without specification as to whether they were asymptomatic or not [19]. Another prospective cohort study investigating the prognostic value of new spinal lesions occurring during the first 3 years after CIS onset did not specifically report the proportion of asymptomatic lesions [20].

Spinal lesions, although generally occurring less frequently compared to brain lesions [17], can develop independently [21]. In our subgroup of 168 patients with coupled spinal and brain MRIs available, 10.12% had a-SL only, in line with the finding from our previous study (10%) [9]. In a cross sectional study involving CIS and RRMS, among 340 spinal MRIs with new T2 or enhancing lesions, 12.1% belonged to asymptomatic patients free from new brain lesions [8]. Taken together, these findings indicate that including spinal MRI in the radiological follow-up of clinically stable MS patients, could unveil disease activity otherwise neglected by brain MRI alone, in approximately 10% of the patients.

The second main result of this study was that the occurrence of a-SL conferred an increased risk of developing new brain demyelinating lesions, and even more recurrent spinal lesions during a median follow up of 31 months. This finding was strengthened by a sensitivity analysis in which the concomitant occurrence of a-BL was accounted for. In this analysis, the presence of either a-SL or a-BL was also predictive of new brain and spinal lesions at follow-up. The prognostic value of a-SL predominantly concerned the future accumulation of spinal rather than brain lesions, and similarly, a-BL better predicted the occurrence of brain lesions. Therefore, our study strengthens the concept that some MS patients appear to have tendency for recurrent spinal involvement up to the extreme clinical picture of the so called “pure spinal MS” [13,22].

In patients with an established MS diagnosis, spinal involvement has been associated with disability accrual [12]. However, the presence of new, longitudinally assessed a-SL in our cohort was not directly associated with disability progression. Similarly, no difference was seen by Dekker et al. during a median follow-up of 6 years in the time to reach EDSS three and six in MS patients with and without accumulation of new spinal lesions at 2 years following disease onset [19]. Brownlee et al., showed instead that accumulation of new spinal lesions at 1 and 3 years following baseline was associated with higher EDSS scores at 15 years in a cohort of 178 patients with CIS, suggesting the relevance of longer term follow-up in evaluating disability outcomes [20]. Notably, studies investigating the presence, rather than the accumulation, of spinal lesions are more suggestive of a role in predicting longer term disability at different stages of MS [11,20,23,24,25]. Given that a-SL was associated in our study with an increased risk of additional spinal cord lesions, and the known association of spinal demyelination with worse clinical outcomes, it would be reasonable to hypothesize that patients with a-SL are at increased risk of disability accrual. However, such association was not present in our study and remains, therefore, speculative.

We found a consistent association between disability worsening and progressive MS subtypes, in line with the observation of a rather stable progression of disability once certain EDSS steps have been reached [26], and with the notion of a reduced therapeutic window in more advanced MS [27]. Additionally, higher EDSS scores were associated with fewer new lesions in spinal MRIs, highlighting that mechanisms underlying more advanced disease are different from focal inflammatory activity. No variables were associated with the risk of relapses in our study, contrarily to our previous findings [9]. We believe this likely reflects the overall lower number of relapses as a consequence to the currently larger availability of high-efficacy MS drugs.

Our study has limitations. Firstly, the retrospective design, which is partially mitigated by the standardized collection of data within our center and local MS registry. Several changes in disease modifying treatments occurred in these patients before study inclusion, between T1 and T2, as well as after T2. It is difficult to disentangle treatment effects in this context. Secondly, this study does not analyze cord atrophy, which has been shown to be independently associated with disability in MS [28]. However, despite having been shown to be promising, atrophy measures are not yet routinely used in the clinical practice. Additionally, because of the limited sample size, we decided not to consider number, location (cervical vs. thoracic) and size of the asymptomatic lesions (e.g., diffuse spinal cord abnormalities), which are pathological features seen prevalently in progressive MS subtypes [29]. Finally, clinical stability was defined by the absence of acute relapses and stable EDSS scores, but subtle changes in neurological function that do not qualify as relapses and do not impact EDSS at time of spinal lesions appearance cannot be excluded.

In conclusion, we found that asymptomatic spinal lesions are detectable in approximately 15% of clinically stable MS patients over a median period of 14 months. If present, a-SL confers an increased risk of future accumulation of brain and spinal demyelinating lesions, particularly at the spinal level, that may contribute to disability accrual over the long term. A link between asymptomatic spinal demyelination and disability worsening remains to be established, as well as whether the appearance of a-SL should prompt changes in disease modifying treatments. Despite this, our data suggest it might be useful to consider spinal MRI in the monitoring of clinically stable MS patients, particularly in case of spinal involvement.

## Figures and Tables

**Table 1:** Baseline demographic and clinical characteristics of all patients included in the study (n = 300), those with also available brain MRI within ±60 days from T1 and T2 (brain MRI-subgroup, n = 168) and those with no available brain MRI within ±60 days from T1 and T2 (n = 132). The *p* value refers to the comparison between patients with vs. without brain MRI within ±60 days from T1 and T2. DMT: disease modifying treatment.

	All Patients Included (n = 300)	Brain MRI Available at T1 and T2 (n = 168)	Brain MRI Not Available at T1 and T2 (n = 132)	*p*-Value
Female sex, n (%)	209 (69.67)	119 (70.83)	90 (68.18)	0.620
Disease course, n (%)				0.048
RRMS	249 (83.00)	132 (78.57)	117 (88.64)	
SPMS	36 (12.00)	24 (14.29)	12 (9.09)	
PPMS	15 (5.00)	12 (7.14)	3 (2.27)	
Age, years, mean (SD)	42.45 (11.87)	42.53 (11.67)	42.35 (12.16)	0.619
Years since MS symptoms onset, mean (SD)	9.81 (8.92)	9.49 (8.83)	10.22 (9.04)	0.774
EDSS score, median (range)	2.5 (0.0–7.0)	2.5 (0.0–7.0)	2.0 (0.0–6.5)	0.166
Interval range t1–t2, median (range), months	14.30 (12.00–20.83)	13.15 (11.70–19.25)	16.03 (11.74–20.10)	0.347
Interval range t2–t3, median (range), months	31.07 (17.14–49.99)	37.28 (19.96–65.57)	23.47 (14.00–39.12)	<0.0001
Treatments, n (%)				0.151
Injectable DMT	110 (36.67)	66 (36.29)	44 (33.33)	
Oral DMT	63 (21.00)	28 (16.67)	35 (26.52)	
Monoclonal antibodies	89 (29.67)	48 (28.57)	41 (30.06)	
Others	17 (5.67)	12 (7.14)	5 (3.79)	
None	21 (7.00)	14 (8.33)	7 (5.30)	
Brain MRI at T1, n (%)				
T2 lesions ≥ 9	296 (98.67)	164 (97.62)	132 (100)	0.133
Spinal MRI at T1, n (%)				
T2 lesions ≥ 1	269 (89.67)	157 (93.45)	112 (84.45)	0.015
Cervical lesions ≥ 1	253 (84.33)	150 (89.29)	103 (78.03)	0.008
Thoracic lesions ≥ 1	194 (64.67)	113 (67.26)	81 (61.36)	0.289

**Table 2:** Univariate Cox regression and Poisson regression models testing variables associated with time to Expanded Disability Status Scale (EDSS) progression, time to first relapse (TTFR) and annualized relapse rate (ARR).

Variable		Disability in All Patients (n = 300)		Relapses in RRMS (n = 249)			
		Time to EDSS Progression		TTFR		ARR	
		HR (95% CI)	*p*	HR (95% CI)	*p*	RR (95% CI)	*p*
a-SL	no	-	-	-	-	-	-
	yes	0.68 (0.29–1.58)	0.367	1.79 (0.67–4.79)	0.240	1.76 (0.75–3.62)	0.154
Age at MS onset	per year	1.01 (0.99–1.04)	0.229	1.01 (0.97–1.04)	0.747	0.99 (0.96–1.02)	0.623
Disease duration	per year	0.99 (0.95–1.02)	0.386	0.99 (0.94–1.04)	0.684	0.98 (0.93–1.02)	0.346
Gender	female	-	-	-	-	-	-
	male	0.85 (0.49–1.49)	0.571	0.82 (0.35–1.96)	0.661	0.83 (0.40–1.62)	0.609
EDSS score	per point	1.01 (0.84–1.20)	0.947	0.75 (0.53–1.05)	0.099	0.69 (0.52–0.91)	0.010
Treatment history	untreated	-	-	-	-	-	-
	treated	0.56 (0.26–1.24)	0.151	1.02 (0.24–4.33)	0.975	0.74 (0.30–2.47)	0.568
Disease course	RRMS	-	-	-	-	-	-
	PPMS	4.33 (2.08–9.02)	<0.0001	-	-	-	-
	SPMS	2.63 (1.41–4.89)	<0.0001	-	-	-	-

**Table 3:** Univariate and multivariate Poisson regression models testing variables associated with occurrence of new brain and spinal MRI lesions at T3 as compared to T2.

Variable		New Brain Lesions at T3				New Spinal Lesions at T3			
		Univariate Analysis		Multivariate Analysis		Univariate Analysis		Multivariate Analysis	
		RR (95% CI)	*p*	RR (95% CI)	*p*	RR (95% CI)	*p*	RR (95% CI)	*p*
a-SL	no	-	-	-	-	-	-	-	-
	yes	1.67 (1.19–2.30)	0.002	1.63 (1.16–2.25)	0.003	7.99 (4.44–14.42)	<0.0001	7.28 (4.02–13.22)	<0.0001
Age at MS onset	per year	0.96 (0.95–0.97)	<0.0001	0.98 (0.97–1.00)	0.024	0.98 (0.95–1.00)	0.096	-	-
Disease duration	per year	0.96 (0.94–0.98)	<0.0001	-	-	0.93 (0.89–0.98)	0.005	-	-
Gender	female	-	-	-	-	-	-	-	-
	male	0.58 (0.42–0.79)	0.001	0.72 (0.52–0.99)	0.046	0.79 (0.39–1.49)	0.491		
EDSS score	per point	0.64 (0.57–0.71)	<0.0001	0.72 (0.63–0.83)	<0.0001	0.70 (0.55–0.88)	0.003	0.74 (0.56–0.96)	0.032
Treatment history	untreated	-	-	-	-	-	-	-	-
	treated	0.81 (0.53–1.33)	0.38	-	-	0.69 (0.30–1.99)	0.430	-	-
Disease course	RRMS	-	-	-	-	-	-	-	-
	Progressive MS	0.37 (0.23–0.58)	<0.0001	-	-	0.51 (0.17–1.17)	0.153	-	-

## Data Availability

Data are available upon request to the corresponding author.

## Supplementary Materials

The following are available online at https://www.mdpi.com/2077-0383/10/3/463/s1, Table S1: Univariate Cox regression testing the association between presence at T2 of a-SL, a-BL, either a-SL or a-BL as well as additional covariates with time to EDSS progression after T2. Table S2: Univariate Cox and Poisson regressions testing within RRMS patients the association between presence at T2 of a-SL, a-BL, either a-SL or a-BL and additional covariates with annualized relapse rate (ARR) between T2 and T3 and time to first relapse (TTFR) after T2. Table S3: Univariate and multivariate Poisson regression testing the association between presence at T2 of a-SL, a-BL, either a-SL or a-BL as well as additional covariates with annualized brain lesion rate between T2 and T3. Table S4: Univariate and multivariate Poisson regression testing the association between presence at T2 of a-SL, a-BL, either a-SL or a-BL as well as additional covariates with annualized spinal lesion rate between T2 and T3.
