# Supplementary material for: Recurrence and Prognostic Value of Asymptomatic Spinal Cord Lesions in Multiple Sclerosis
Source: J Clin Med. 2021 Jan 26;10(3):463. doi: 10.3390/jcm10030463 (PMC7865947; doi:10.3390/jcm10030463)
Supplement: Supplementary file 1 [file jcm-10-00463-s001.pdf]

SUPPLEMENTARY MATERIAL

MRI protocols

Spinal cord: a) sagittal proton density (PD) weighted turbo-spin-echo (TSE) of the cervical and thoracic segments (two separate acquisitions) (repetition time (TR)=2000 ms, echo time (TE)=9 ms, slice thickness=3 mm, slice gap = 10%; in-plane voxel size=0.42x042mm^2); b) axial T2-weighted multi-echo gradient echo in the cervical segment (TR=510 ms, TE=14 ms, slice thickness=3 mm, slice gap=10%, in-plane voxel size=0.4x0.4mm^2) and TSE in the thoracic segment (TR=3500 ms, TE=103 ms, slice thickness=3 mm, slice gap=10%, voxel size=0.31x0.31mm^2).

Brain: a) axial dual-echo TSE (TR=2670 ms, TE=24/120 ms, slice thickness=3 mm, gap=0%, acquisition parallel to the AC-PC plane, voxel size=0.98x0.98x3.3 mm^3); and b) sagittal 3D T1-weighted MPRAGE (TR=2300 ms, TE=2.81 ms, FA=9°, TI=900 ms, matrix=256x256, slice thickness=0.9 mm, slice number=192, voxel size=0.93x0.93x0.9 mm^3).

Supplementary tables

Supplementary table 1: Univariate Cox regression testing the association between presence at T2 of a-SL, a-BL, either a-SL or a-BL as well as additional covariates with time to EDSS progression after T2.

**Variables**	**Time to EDSS progression**	
	**HR (95%CI)**	**p-value**
**a-SPL**	1.00 (0.42-2.38)	0.995
**a-BL**	1.23 (0.57-2.67)	0.593
**a-BL or a-SPL**	1.14 (0.59-2.24)	0.693
**Age at MS onset, years**	1.03 (1.00-1.05)	0.052
**Disease duration, years**	0.98 (0.94-1.02)	0.356
**Gender (male vs female)**	1.08 (0.57-2.03)	0.815
**EDSS score**	1.00 (0.82-1.22)	0.996
**Treatment history (previously treated vs naive)**	0.58 (0.24-1.39)	0.223
**Relapsing Remitting (as reference)**	-	
Primary Progressive	3.98 (1.72-9.21)	0.001
Secondary Progressive	2.31 (1.11-4.79)	0.001

Supplementary table 2: Univariate Cox and Poisson regressions testing within RRMS patients the association between presence at T2 of a-SL, a-BL, either a-SL or a-BL and additional covariates with annualized relapse rate (ARR) between T2 and T3 and time to first relapse (TTFR) after T2.

**Variables**	**ARR**		**TTFR**	
	**RR (95%CI)**	**p-value**	**HR (95%CI)**	**p-value**
**a-SPL**	0.80 (0.17-2.76)	0.757	0.83 (0.19-3.62)	0.800
**a-BL**	1.42 (0.49-3.78)	0.514	1.35 (0.44-4.14)	0.597
**a-BL or a-SPL**	1.15 (0.42-2.91)	0.780	1.12 (0.40-3.18)	0.824
**Age at MS onset, years**	1.00 (0.96-1.04)	0.967	1.02 (0.97-1.06)	0.437
**Disease duration, years**	0.98 (0.91-1.04)	0.500	0.99 (0.93-1.06)	0.841
**Gender (male vs female)**	0.80 (0.28-2.11)	0.675	0.94 (0.34-2.65)	0.914
**EDSS score**	0.69 (0.46-1.00)	0.056	0.83 (0.56-1.23)	0.350
**Treatment history (previously treated vs naive)**	1.00 (0.26-4.98)	0.997	2.04 (0.27-15.38)	0.488

Supplementary table 3: Univariate and multivariate Poisson regression testing the association between presence at T2 of a-SL, a-BL, either a-SL or a-BL as well as additional covariates with annualized brain lesion rate between T2 and T3.

**Variables**	**Univariate analysis**		**Multivariate analysis**	
	**RR (95%CI)**	**p-value**	**RR (95%CI)**	**p-value**
**a-SPL**	1.38 (0.85-2.14)	0.169		
**a-BL**	1.66 (1.08-2.48)	0.016		
**a-BL or a-SPL**	2.00 (1.39-2.84)	0.0001	1.81 (1.25-2.60)	0.001
**Age at MS onset, years**	0.96 (0.94-0.97)	<0.0001	0.97 (0.95-0.99)	0.009
**Disease duration, years**	0.97 (0.94-0.99)	0.008		
**Gender (male vs female)**	0.60 (0.39-0.88)	0.012		
**EDSS score**	0.67 (0.58-0.77)	<.0001	0.80 (0.67-0.95)	0.012
**Treatment history (previously treated vs naive)**	1.36 (0.73-2.88)	0.378		
**Disease phase (RRMS vs progressive)**	0.28 (0.14-0.50)	0.0001	0.53 (0.24-1.03)	0.076

Supplementary table 4: Univariate and multivariate Poisson regression testing the association between presence at T2 of a-SL, a-BL, either a-SL or a-BL as well as additional covariates with annualized spinal lesion rate between T2 and T3.

**Factors**	**Univariate analysis**		**Multivariate analysis**	
	**RR (95%CI)**	**p-value**	**RR (95%CI)**	**p-value**
**a-SPL**	5.45 (2.64-11.04)	<0.0001		
**a-BL**	2.01 (0.84-4.31)	0.089		
**a-BL or a-SPL**	3.08 (1.51-6.26)	0.002	2.63 (1.27-5.45)	0.009
**Age at MS onset, years**	0.98 (0.94-1.01)	0.181		
**Disease duration, years**	0.94 (0.87-0.99)	0.044		
**Gender (male vs female)**	1.02 (0.46-2.11)	0.961		
**EDSS score**	0.68 (0.52-0.89)	0.007	0.74 (0.53-1.00)	0.059
**Treatment history (previously treated vs naive)**	1.02 (0.36-4.27)	0.972		
**Disease phase (RRMS vs progressive)**	0.53 (0.16-1.36)	0.238		
